# The Contribution of Scanning Force Microscopy on Dental Research: A Narrative Review

**DOI:** 10.3390/ma17092100

**Published:** 2024-04-29

**Authors:** Christine Müller-Renno, Christiane Ziegler

**Affiliations:** Department of Physics and Research Center OPTIMAS, RPTU Kaiserslautern, 67663 Kaiserslautern, Germany; cz@physik.uni-kl.de

**Keywords:** scanning force microscopy, scanning force spectroscopy, single-cell force spectroscopy, dental materials, dental research, pellicle, dental biofilm

## Abstract

Scanning force microscopy (SFM) is one of the most widely used techniques in biomaterials research. In addition to imaging the materials of interest, SFM enables the mapping of mechanical properties and biological responses with sub-nanometer resolution and piconewton sensitivity. This review aims to give an overview of using the scanning force microscope (SFM) for investigations on dental materials. In particular, SFM-derived methods such as force–distance curves (scanning force spectroscopy), lateral force spectroscopy, and applications of the FluidFM^®^ will be presented. In addition to the properties of dental materials, this paper reports the development of the pellicle by the interaction of biopolymers such as proteins and polysaccharides, as well as the interaction of bacteria with dental materials.

## 1. Introduction

Dental materials are pivotal in modern dentistry, encompassing various substances to restore, repair, and maintain oral structures. Dental materials are broadly categorized into natural enamel, restorative, prosthetic, and auxiliary materials, each serving specific functions in dental treatment modalities. Restorative materials such as dental amalgam, composite resins, and glass ionomers are utilized for repairing and restoring damaged teeth, providing durability and biocompatibility [1,2,3]. Prosthetic materials, including ceramics, metals, and polymers, are employed for fabricating dental prostheses such as crowns, bridges, and dentures, giving structural support, functionality, and aesthetics [1,3,4]. Auxiliary materials such as impression materials, cement, and bonding agents play essential roles in dental procedures by facilitating accurate impressions, cementation, and adhesion of dental restorations [1,2,3]. The properties of dental materials, including mechanical, physical, chemical, and biocompatibility characteristics, are critical factors influencing their clinical performance and longevity. Advancements in dental material science have led to innovative materials with enhanced properties, such as bioactive ceramics, nanocomposites, and tissue-engineered constructs, paving the way for improved clinical outcomes and patient care.

Scanning force microscopy (SFM), also called atomic force microscopy (AFM) in the literature, has emerged as a versatile and powerful tool in the field of biological, biomaterial, and dental materials research, offering unprecedented capabilities to investigate the intricate interactions from biopolymers to bacteria with dental substrates at the nanoscale [5,6]. In dental health, understanding the complex interplay between bacteria and dental materials is crucial for developing strategies to prevent biofilm formation, dental caries, and periodontal diseases.

In this review, we explore the application of SFM (also combined with the FluidFM^®^, fluidic force microscopy, Cytosurge, Opfikon, Switzerland) in visualizing dental materials, characterizing their nanomechanical properties, and elucidating the dynamic interactions between biopolymers, bacterial biofilms, and dental materials. Recent combinations of SFM with other techniques, such as infrared spectroscopy (IR), Raman spectroscopy, laser scanning microscopy, and fluorescence microscopy, are beyond the scope of this review. Interested readers are referred to [7,8,9,10].

SFM enables researchers to visualize and quantify surface topography, and mechanical and adhesion properties of dental substrates in real time, providing valuable insights into the erosion of dental materials and the mechanisms underlying bacterial adhesion, colonization, and biofilm formation on dental surfaces. By employing SFM techniques, researchers can characterize materials characteristics like surface roughness, chemistry, and morphology of dental materials as well as their influence on bacterial adhesion and biofilm accumulation, facilitating the development of novel antimicrobial coatings, surface modifications, and therapeutic strategies to mitigate bacterial colonization and enhance the biocompatibility of dental biomaterials. Furthermore, SFM offers unique capabilities to study the nanoscale interactions between biopolymers, bacterial adhesins, and extracellular polymeric substances (EPS) within biofilms, shedding light on the molecular mechanisms of bacterial attachment, biofilm maturation, and resistance to antimicrobial agents. We will delve into the principles of SFM and its applications in imaging and characterizing dental materials. In addition, we will explore recent advancements and challenges in utilizing SFM as a powerful tool for understanding and controlling biopolymer, respectively, bacterial interactions with dental substrates, ultimately contributing to developing innovative strategies for improving oral health and dental care.

For the narrative review, we considered the full-time period of the SFM, beginning in the early 1990s and ending today. The number of available publications on the topic is so huge that we could not cite them all here. However, we have used the publications (only journal articles and reviews) that include the most representative and important applications.

## 2. Instruments, Operation Modes and Methods

Scanning force microscopy was invented in 1986 by Binnig, Quate, and Gerber and originated from the scanning tunneling microscope [11]. A scanning probe, the cantilever with a sharp tip, is used to map a surface at the atomic level.

### 2.1. SFM Principle

**Figure 1:**
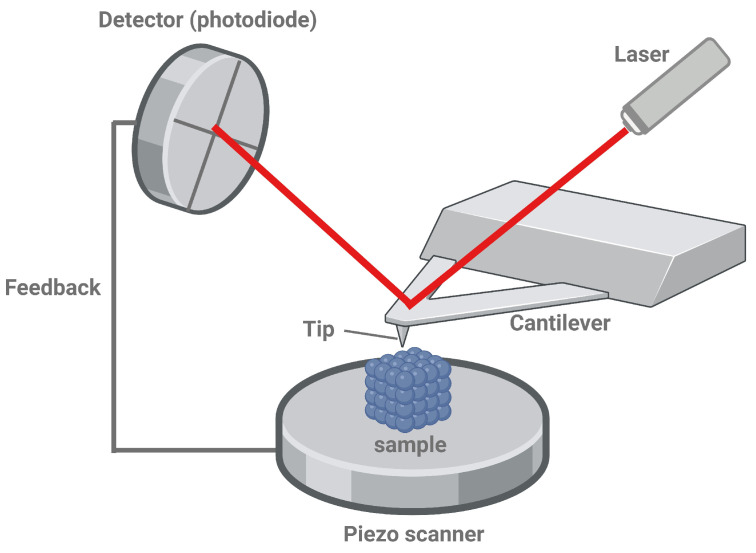
Principle of SFM. Created with BioRender.com.

Unlike optical microscopes, SFM does not use a light beam combined with glass lenses to create an image of the sample. A tip (probe) on a more or less flexible cantilever is used, which scans the sample. The tip interacts with the force field near the surface, leading to a deflection of the cantilever beam. The latter is detected using a laser according to the light pointer principle together with a four-segment photodiode (see Figure 1). The image resolution strongly depends on the sharpness of the used tip. Therefore, resolutions down to the sub-nm regime are possible (in contrast to optical microscopes, which are limited to a fraction of the used wavelength).

Compared to the Scanning Electron Microscope (SEM), SFM may have a limitation in scan area size. However, technological advancements, such as motorized sample scanner stages, now allow for measuring challenging biological samples up to 20 × 20 mm^2^. On the other hand, SEM has drawbacks, such as the requirement for liquid-free environments, the need for conductive samples, and limited height information (only 2D data).

The SFM operates in a feedback loop mechanism to maintain a constant force of interaction between the probe tip and the sample surface. This constant force is achieved by continuously adjusting the probe’s position to keep the controlled variable constant. This feedback loop ensures accurate topographical 3D mapping of the sample surface.

**Figure 2:**
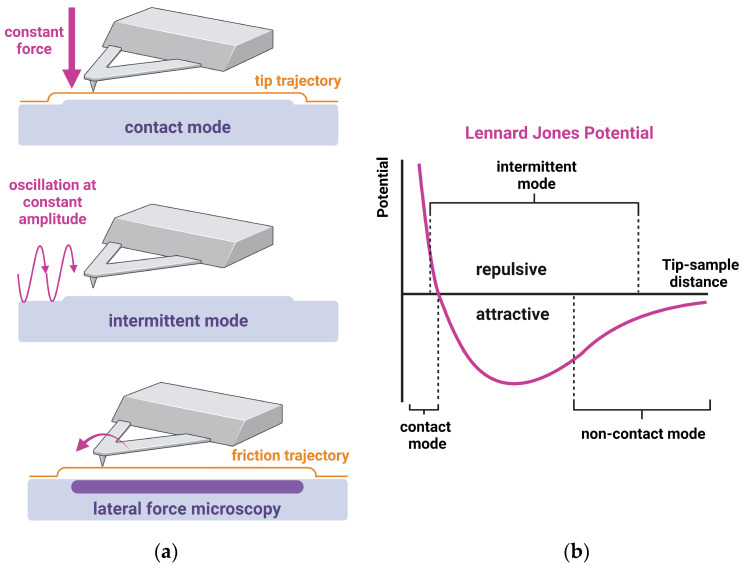
(**a**) Sketch of the most prominent SFM imaging modes in dental application. (**b**) Lennard–Jones potential, describing the tip–sample interaction. Created with BioRender.com.

At the heart of SFM, thus, lies the intricate interplay of various forces that enable precise imaging and characterization of sample surfaces. These forces include van der Waals forces, electrostatic forces, and capillary forces, among others. Van der Waals forces are one of the fundamental forces in SFM. These forces arise due to the interaction between electrically neutral atoms and molecules. The Lennard–Jones potential (see Figure 2b) provides a theoretical framework for understanding the van der Waals interaction between the SFM probe tip and the atoms or molecules on the sample surface. At larger distances, the attractive component of the Lennard–Jones potential leads to adhesion between the probe tip and the sample surface. Conversely, at shorter distances, the repulsive component of the Lennard–Jones potential comes into play, preventing the probe tip from penetrating the sample surface.

When a charged probe tip approaches a charged surface, electrostatic forces come into play, leading to either attraction or repulsion between the tip and the sample.

Capillary forces can lead to adhesion between the tip and the sample, affecting the accuracy of SFM measurements. These forces arise due to the surface tension of liquids present between the SFM probe tip and the sample surface. Therefore, it makes sense to control environmental conditions depending on the sample and the humidity to minimize the impact of capillary forces on SFM imaging.

Additionally, other forces such as magnetic forces, chemical bonding forces, and mechanical forces may also influence SFM measurements depending on the specific experimental setup and sample properties.

For most purposes, only the van der Waals forces should act on the cantilever. The other main interaction forces such as Coulomb or capillary forces have to be suppressed by appropriate methods. This is normally the case if biological samples are measured in a buffer solution. The ions shield the Coulomb forces, and no capillary forces exist for an immersed cantilever. However, great care has to be taken that the ionic strength of the solution is kept constant during experiments.

### 2.2. SFM Operating and Imaging Modes

SFMs can operate in various scanning modes, each offering unique capabilities (see Figure 2a). The most common modes include the contact, intermittent, and non-contact modes.

In the contact mode, the tip is physically in contact with the sample. It can be run in the constant force and constant height modes. In the constant force mode, the force acting between the tip and the sample is kept constant and is controlled by a feedback system. The distance between cantilever fixation and the sample is kept constant during the constant height mode. This mode runs without any feedback system, which delivers different magnitudes of force acting on the cantilever.

In the contact mode, the scanning motion of the cantilever relative to the substrate leads to a lateral torsion of the cantilever, reflecting the frictional properties of the sample. This kind of measurement is called lateral force microscopy (LFM).

To reduce frictional interactions and damage of, e.g., biological samples, the dynamic modes (intermittent and non-contact) are available. In both dynamic modes, the cantilever oscillates. In the non-contact mode, the cantilever oscillates near its resonance frequency, and the tip has no contact with the substrate. This mode uses long-range forces and can only be used in a vacuum, making it less useful for most biological problems. The intermittent mode, however, combines an oscillating cantilever with the contact mode. The cantilever oscillates, and the tip touches the substrate in each oscillation, accompanied by a loss of amplitude. This amplitude loss is used in the feedback to reach the target amplitude again and to image topographical features. The intermittent mode uses the repulsive and the attractive forces between the tip and the sample.

### 2.3. Force–Distance Measurements

**Figure 3:**
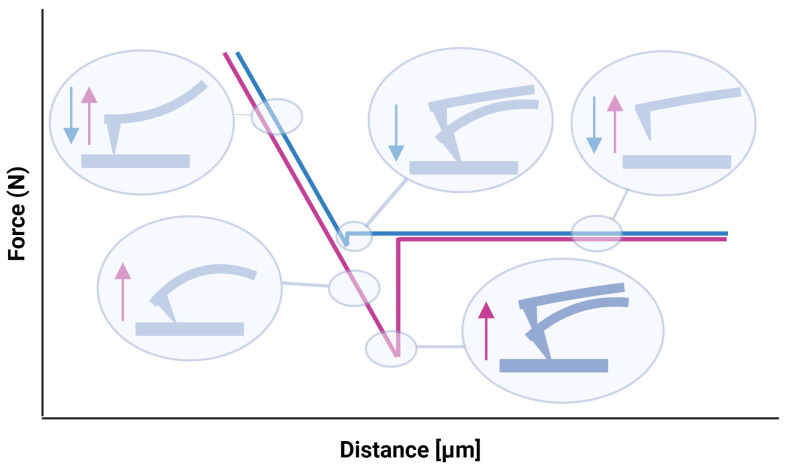
Exemplary force–distance curve. Blue: trace; red: retrace. Created with BioRender.com.

Force–distance measurements by scanning force microscopy (SFM) are crucial in understanding surface properties and interactions at the nanoscale. This technique involves probing the sample surface with a sharp tip attached to a cantilever while simultaneously measuring the force exerted between the tip and the surface as a function of their separation distance. Figure 3 represents an exemplary force–distance curve. Initially, the tip is positioned far away from the sample surface, and the cantilever is in its neutral position. As the tip approaches the surface, the cantilever begins to deflect due to attractive or repulsive forces between the tip and the sample. The cantilever deflection is converted into force using calibration procedures, which typically involve measuring the cantilever’s spring constant and applying Hooke’s law to relate deflection to force [6,12,13,14]. After reaching a certain point of interaction, the tip is retracted from the surface. During this retraction, the cantilever deflection changes as the tip–sample interaction diminishes. The resulting retraction curve provides information about the adhesive forces of the sample. The approach curve contains the elastic properties of the sample. The area between the approach and retraction curve gives insights into the adhesion work. These measurements are vital for characterizing materials, understanding surface phenomena, and interaction forces in various fields such as materials science, biology, and nanotechnology.

Based on force volume measurements, quantitative imaging modes (Peak Force QNM™ and QI Mode™) have been developed over the last ten to fifteen years [15,16,17]. A virtual grid covers the entire scanning range, with a force–distance curve measured at each grid point. Following this, software functionalities analyze the one-dimensional data from the force curves. These results are then integrated with the two-dimensional grid data to generate a three-dimensional map. In addition to topographical details, typically found in standard imaging modes, these data contain an image of elasticity, adhesion between the tip and sample, and adhesion work. The impact of lateral forces on the sample and tip damage is not present in these modes.

## 3. Material Aspects of Dental Materials

SFM has emerged as a groundbreaking technique in the field of dentistry research, offering unparalleled capabilities for imaging and characterizing dental materials from natural enamel to artificial dental materials at the nanoscale. One of the key advantages of SFM is its ability to capture three-dimensional images of dental materials with unprecedented detail, allowing for the visualization of nanometer-scale features often inaccessible through traditional microscopy techniques. SFM provides valuable insights into various dental substrates’ surface topography, morphology, and mechanical properties, including natural enamel, ceramics, composites, polymers, and metals. By employing SFM, researchers and practitioners can precisely analyze the nanoscale features of dental materials, such as surface roughness, wear patterns, and interface properties, which are critical for understanding their performance and durability in clinical settings.

**Figure 4:**
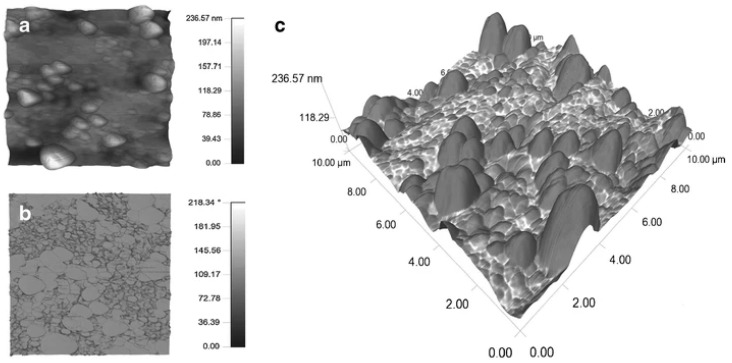
SFM image of the enamel surface. (**a**) A 10 µm height image which contains the topographic information. (**b**) A 10 µm phase image in which chemical differences determine the contrast. * of the scale bar refers to phase shift (**c**) Overlay of height (**a**) and phase (**b**) image revealing enamel prisms (dark) and proteinaceous regions (bright). Reproduced with permission from Springer [22].

Individual crystals up to prisms were observed on (human) enamel surfaces by scanning force microscopy [18,19,20] and compared to animal teeth structures [21]. Figure 4 represents an image of natural enamel taken in the dynamic mode, as an overlay of height and phase. In particular, the phase (shift between the oscillating piezo driving force and the cantilever response on the photodiode and thus a loss signal) reveals the enamel prisms.

In addition to imaging dental materials in static conditions, SFM can also be utilized to study dynamic processes such as wear, degradation, and biofilm formation in real time. By monitoring changes in surface topography and mechanical properties over time, SFM enables researchers to assess dental materials’ long-term stability and performance in simulated oral environments, providing valuable data for optimizing their design and composition.

**Figure 5:**
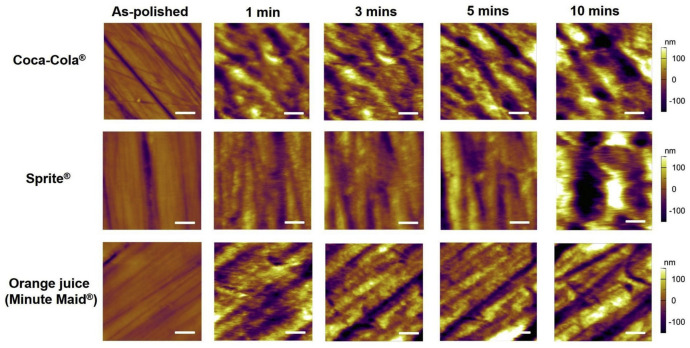
Variation in the enamel surface topography with different immersion times treated with Coca-Cola^®^, Sprite^®^, and orange juice (Minute Maid^®^). Scale bars 2 µm. Reproduced with permission from Science Direct [23].

Li et al. presented the nanoscale effects of beverages on human enamel surfaces [23]; see Figure 5. They evaluated the mechanical and morphological changes in enamel surfaces induced by soft drinks, noting a significant increase in surface roughness with increasing immersion time. After 1 h of immersion, the prismatic structure of the enamel became visible. Additionally, nanomechanical characterization revealed a considerable decrease in elastic modulus as the soft drinks etched away enamel structures [23]. The increased surface roughness of the enamel surface is believed to facilitate easier bacterial adhesion.

Finke et al. conducted comparable studies on the early stages of food-induced native enamel dissolution [24]. They investigated the effects of mineral water, a blackcurrant drink, and lemon and lime juice at different exposure times, demonstrating that SFM is useful for measuring the early stages of enamel surface dissolution. Watari showed similar effects for different acid agents, such as citric and phosphoric acid [25]. They observed a linear increase in etching up to 3 min of etching time, with different acid agents exhibiting varying demineralization power, etching rate, and morphology [25]. Further studies by Segota [26] also support these findings. Additionally, SFM revealed the differences between healthy and fluorotic enamel [26]. Zavalo-Alonso et al. characterized the external structure, roughness, and absolute depth profile of fluorotic and healthy enamel, confirming a positive association between fluorosis severity, enamel surface roughness, and absolute depth profile at the nanometer scale [27]. Furthermore, Zeitz et al. and Faidt et al. demonstrated increased resistance of hydroxyapatite against acids due to fluorination of the material [28,29,30]. Furthermore, Hannig et al. successfully utilized the contact mode of SFM to image and identify pellicle structures on enamel [31,32]. The adsorbed salivary pellicle was visualized as a dense, rounded structure.

In addition to the mentioned studies on natural enamel surfaces, SFM also found applications in studying artificial dental or dental implant materials [33,34,35]. Here, the focus was on nanocharacterization and quantification of changes in the physicochemical properties of dental alloys after processing [26]. Even in the characterization of novel dental materials after glazing and polishing, the SFM finds its application [36].

**Figure 6:**
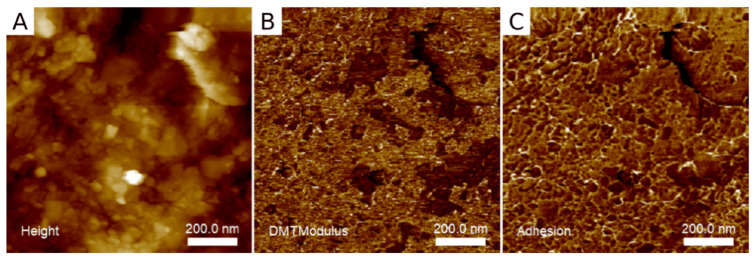
Topography (**A**), Young’s Modulus (**B**), and adhesion (**C**) of unetched natural enamel mapped with Peak Force QNM. Reproduced with permission from Machoy et al. [37].

In addition to imaging, SFM provides deep insights into the nanomechanical properties of the samples under investigation. Machoy et al. could map the topography, Young’s Modulus, and the adhesion of natural enamel substrates using the quantitative image mode Peak Force QNM™ [37]. The authors correlated the topographic images with the nanomechanical properties. They found that etching increases the enamel roughness and reduces the hardness, while resin coverage reduces the roughness and increases the hardness. However, the highest hardness showed intact enamel. Figure 6 shows exemplary topography, Young’s Modulus, and adhesion images. Alharbi et al. recently published a quantitative nanomechanical mapping on novel dental materials (model polymer infiltrated ceramic network materials compared to CAD/CAM resin composite blocks) using Peak Force QNM™ mode (see Section 2.3). The authors investigated the roughness and elastic modulus of the dental materials and found a significant effect of sintering temperature on topography and nanomechanical properties [38].

Habelitz et al. combined SFM with a nano-indentation technique to image the substrate and determine the mechanical properties of individual enamel rods at various orientations [39]. They discovered a relationship between elasticity/hardness and microstructural texture, attributing the observed enamel anisotropy to the alignment of fiber-like apatite crystals and the composite nature of enamel rods [39]. Similarly, Jeng et al. conducted comparable studies demonstrating an exponential decay of nano hardness and elastic modulus from the enamel surface toward the dentin–enamel junction [40].

Indeed, the scanning force microscope (SFM) offers a versatile platform not only for investigating hard materials, protein layers, bacteria, or cells but also for obtaining ultrastructural insights into biological specimens. While the organism studied by Herrmann, *Caenorhabditis elegans*, may not be directly relevant to dental research, it serves as a remarkable demonstration of the potential of SFM when appropriate sample preparation techniques are employed [41]. Herrmann provided valuable insights into the internal features of *Caenorhabditis elegans* by employing SFM. By utilizing an approach involving polyethylene glycol embedding, ultra-sectioning, and dehydration of the specimen, Herrmann showed the ability of SFM to image the ultrastructural details of virtually any biological material [41]. While the focus may not always be on dentally relevant organisms, the techniques and insights gained from such studies contribute to a broader understanding of biological systems and pave the way for innovative applications in various fields of science and medicine.

## 4. Adhesion Forces (Scanning Force Spectroscopy) on Dental Materials

Proteins and bacteria are the most prominent biomolecules and cells that adhere to oral materials and establish a biofilm [42]. However, polysaccharides also play a role but are underrepresented in research. In addition, different physicochemical surface characteristics can impact bioadsorption in the oral cavity. A lot of effort was made by investigating surfaces with modulated physicochemical properties such as periodic topographies, roughness, surface free energy, or hardness [43,44,45]. Furthermore, micro- and nanostructures, liquid-repellent surfaces, and superhydrophilic/superhydrophobic surfaces were investigated [43,46]. Recently, chemically textured interfaces gained increasing interest and could represent promising solutions for anti-bioadhesive interfaces [45].

The SFM (scanning force microscopy) empowers researchers to delve into the interaction between dental materials and biological entities such as tissues, fluids, molecules, and bacteria [5,47]. By examining adhesion forces at the interface between dental materials and biological environments, SFM aids in developing biomimetic materials with enhanced biocompatibility and performance. By exploring the interface between the implant surface and biological tissues, SFM allows researchers to quantify adhesive forces involved in osseointegration, the process through which the implant integrates with surrounding bone tissue, or pellicle and biofilm formation [5,31,48]. Such information is crucial for optimizing dental implants’ design and surface characteristics to improve their biocompatibility and long-term efficacy. Regarding bacterial adhesion to dental surfaces, SFM provides insights into mechanisms underlying bacterial colonization, biofilm formation, and caries development [31,49,50,51,52]. By functionalizing SFM probes with specific biomolecules or bacterial adhesins, researchers can investigate adhesion forces, aiding in developing antimicrobial coatings and surface modifications to hinder bacterial attachment and biofilm formation [53,54,55,56,57,58,59,60,61,62,63,64,65].

Moreover, the FluidFM^®^ enables researchers to measure adhesion forces between individual bacterial cells and dental materials with exceptional sensitivity without requiring chemical functionalization of the SFM probe. The authors present options for connecting molecules or cells to the cantilever (Section 4.1), followed by a summary of research questions and results regarding the interaction of molecules and bacteria with dentally relevant surfaces (Section 4.2).

### 4.1. Attachment of Molecules and Cells to the SFM Tip

To measure the adhesion forces of biomolecules or cells/bacteria on surfaces, they must be immobilized on the cantilever. Specific and nonspecific binding methods can be employed for this purpose, with specific methods being a more robust option, allowing for the measurement of higher adhesion forces. Dentally relevant biomolecules include proteins and polysaccharides, while bacteria relevant to caries are particularly important in the case of cells.

For protein immobilization, the peptide bond is typically utilized. NH_2_ or COOH groups are presented on the cantilever, and the proteins are covalently attached via COOH or NH_2_ groups. Depending on the research question, it must be determined whether the immobilized proteins should have the ability to rotate and move more freely or whether they should be immobilized more rigidly. In the former case, it is advisable to use a PEG crosslinker [56], which is available with a variable chain length. A zero-length crosslinker via carbodiimide chemistry can be employed in the latter case. EDC (1-ethyl-3-(3-dimethylaminopropyl)carbodiimide) or DCC (Dicyclohexylcarbodiimid) are building a peptide bond and are often used for this purpose, as well as the streptavidin-biotin interaction, Ni-NTA in interaction with His-tagged proteins, and lysine-aldehyde coupling [20,56,57,58]. For polysaccharides, Ehnert et al. successfully coupled dextran covalently to the SFM tip using a photochemical modification [59].

**Figure 7:**
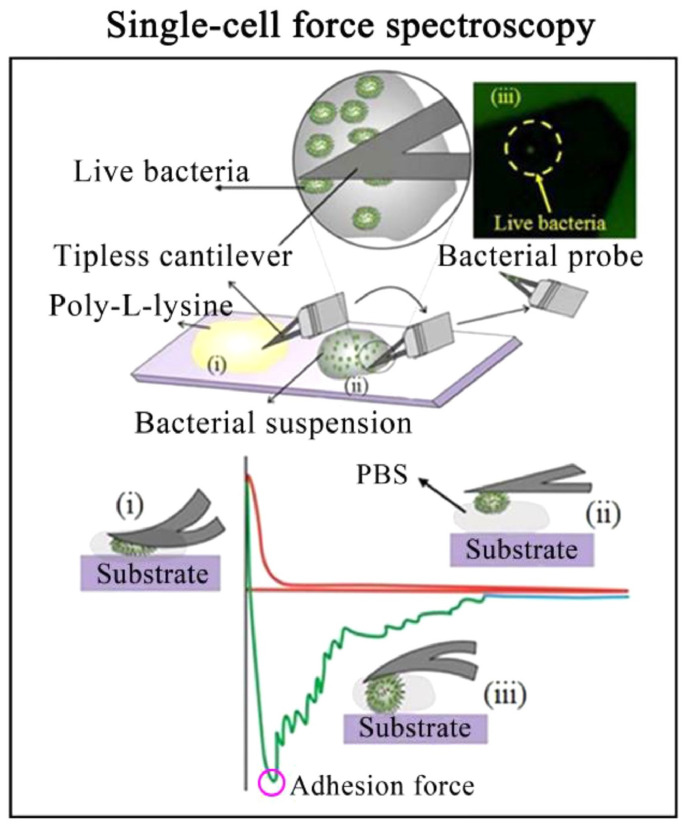
The cartoons show the preparation process of a bacterial probe using poly-l-lysine (**top image**) and the measurement process of single-cell force spectroscopy (SCFS) (**bottom image**). Reproduced with permission from Science Direct [64].

The interaction of cells with surfaces is typically measured with a single cell on the cantilever, termed single-cell force spectroscopy; see Figure 7 [58,59,60,61]. Various chemical options are available for immobilizing cells. While Gram-negative bacteria can usually be immobilized to the cantilever via concanavalin A, polydopamine, poly-l-lysine, or CellTak™ [62], the methodology for Gram-positive bacteria is more challenging. Hofherr et al. examined nine common methods for chemically binding biological molecules/cells to the cantilever regarding binding efficiency [63]. They found significant differences between the various immobilization agents and the parameters used.

To address the efficiency problem in immobilizing bacteria at the cantilever, the FluidFM^®^ represents an ideal solution [65]. The FluidFM combines traditional scanning force microscopy SFM with microchannelled cantilevers connected to a pressure controller. By applying underpressure in the fluidic channel, bacteria (or other cells) can be tightly attracted to the aperture of the tip [66,67,68]. Hofherr et al. compared FluidFM-based single-cell force spectroscopy to conventional bacterial probe SFM and found comparable results for both approaches. They could even successfully conduct adhesion measurements with bacteria that had previously been difficult to immobilize [67].

### 4.2. Adhesion of Biomolecules and Cells to Dentally Relevant Materials

Examining adhesion forces becomes feasible once biomolecules and cells have been successfully connected to the SFM tip. There are virtually no limits regarding materials for investigation, as natural and dental replacement materials and implants can be studied.

**Figure 8:**
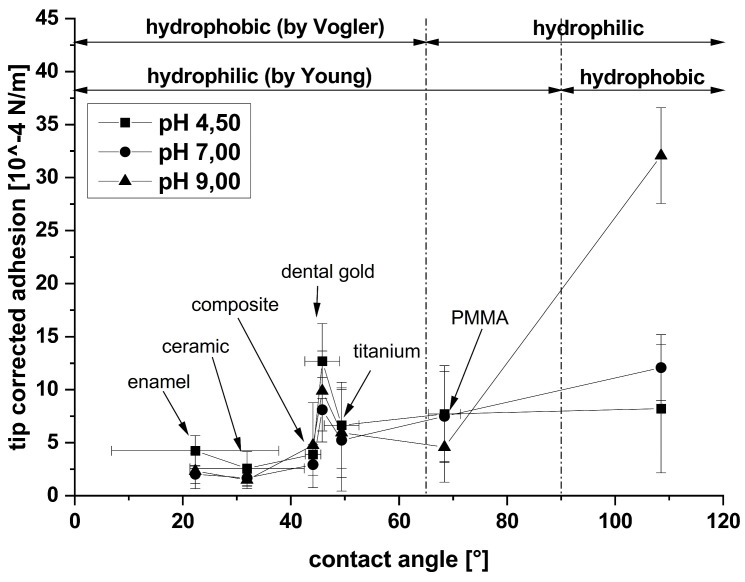
Mean values of the measured adhesion force between BSA and the investigated dental materials (natural enamel and the restorative materials ceramic, composite, dental gold, dental titanium, PMMA, PTFE) as a function of their wettability and solution pH. The connection lines are only guides to the eye. Reproduced with permission from ACS Publications [20].

Müller et al. investigated the adhesion of BSA (coupled to the cantilever using DCC) as a model protein relevant to the pellicle on natural bovine tooth enamel compared to dental replacement materials such as ceramic, composite, gold, titanium, PMMA, and PTFE. Interestingly, natural tooth enamel exhibited the lowest adhesion, while only ceramic and composite showed comparable values to tooth enamel [20]. The authors established a connection between surface wettability and measured adhesion. Additionally, Müller et al. explored the influence of pH value and demonstrated a significant role in electrostatics [20]. The main findings are presented in Figure 8.

Furthermore, Müller et al. determined the adsorbed amount of BSA (using dynamic contact angle analysis) on the above-mentioned dental substrates, concluding that the adsorbed amount correlates well with adhesion forces by SFS on dental materials if no hydrophobic material is involved [22]. Vukosavlijevic et al. also examined the adhesion of pellicle-relevant proteins, i.e., histatin 5, and human serum albumin, to hydroxyapatite using SFM. They utilized biotin-streptavidin-specific binding to immobilize the proteins on the cantilever. The authors observed a substantial difference in measured adhesion and proposed this as a basis for designing synthetic peptides and proteins with protective and therapeutic benefits [69]. Schwender et al. published a comparison of adhesion forces of BSA on natural enamel and Dyract AP, a filling material [70]. They also noted an influence of pH value and a pronounced difference between natural enamel and the filling material (Dyract AP exhibited an increased adhesion force by a factor of 3–3.5). Instead of single proteins, Zhang et al. measured the adhesion of the whole salivary pellicle to human tooth enamel and human tooth enamel covered with the salivary pellicle [71]. They demonstrated that the adhesion strength between the initial salivary pellicle and enamel surface was much higher than between the initial salivary pellicle-covered tip and the substrate. Electrostatic interaction contributed to the adhesion between the initial salivary pellicle and enamel surface but not between the initial salivary pellicle-covered surfaces [71].

After Ehnert et al. successfully covalently attached dextran to the cantilever as described above [59], Link et al. published dextran adhesion data on dental titanium and gold [72]. The authors demonstrated that electrostatics had no significant influence on the adhesion forces due to the lack of charge on the dextran molecule. However, the material itself led to varying levels of adhesion. Link et al. showed that the adhesion of dextran could contribute to biofilm formation on dental materials, which in turn could be crucial for the development of caries and periodontal disease.

After saliva components are bound to dental materials, a so-called conditioning film, the pellicle, is formed, facilitating the subsequent attachment of bacteria. Oral bioadhesion processes and the subsequent bacterial biofilm formation are fundamental to the development of caries and periodontitis. The initial oral biofilm, the pellicle, plays a crucial role in this process. Despite possessing various protective properties, the pellicle is a foundation for bacterial colonization and subsequent biofilm formation on hard tooth surfaces. It’s important to note that bacterial adhesion to orally exposed solid surfaces cannot be entirely prevented. However, new prophylactic preparations aim to delay or reduce bacterial adherence to the tooth surface. One strategy involves making bacterial attachment more difficult and countering the formation of an organized pathogenic biofilm. Scientists seek to understand bacterial adhesion by studying it on dentally relevant surfaces to achieve this goal. As previously explained with proteins and polysaccharides, SFM is the method of choice for such investigations. By connecting individual (single-cell force spectroscopy SCFS; see above Section 4.1) or multiple bacteria (scanning force spectroscopy SFS) to the cantilever, researchers can systematically quantify adhesion forces and examine the influencing parameters of relevant factors.

Spengler et al. showed by single-cell force spectroscopy that *Streptococcus mutans*, the lead organism of dental caries in almost every person’s saliva, is well adapted to the oral cavity, their natural environment. *Streptococcus mutans* showed a pronounced increase in adhesion strength on hydroxyapatite after saliva exposure [49]. The fact that rupture length and detachment work were notably enlarged allowed the conclusion that especially long macromolecules (either from the saliva or produced by the bacteria) contribute to the interaction of *S. mutans* with pellicle-covered hydroxyapatite substrates. In addition, they compared the measurements with the adhesion of a non-caries-relevant bacterium. They found that adapting to the salivary environment is a particular property of *S. mutans* cells [49]. Loskill et al. showed that fluoride has a cariostatic effect in addition to decreasing demineralization [73]. The authors tested the adhesion on the single bacterial cell level of different relevant bacteria species on hydroxyapatite with and without fluoridation. All bacteria species showed reduced adhesion forces after fluoride treatment [73]. Mischo et al. investigated bacterial adhesion by single-cell force spectroscopy to enamel and hydroxyapatite substrates. They showed the possibilities and limitations of a model surface. Through single-cell force spectroscopy, they measured bacterial cell wall proteins’ adhesion force and rupture length after binding to hydroxyapatite and enamel. In addition, they showed the influence of blood plasma and saliva on bacterial adhesion properties. They found that the results match water wettability, the elemental composition of the samples, and the change in the macromolecules adsorbed over time on the surface. Their studies supported the idea that hydroxyapatite is a good alternative to natural dental material. In addition, they found that any pre-adsorbed protein film reduces the bacterial adhesion strength [50]. In recent years, multi-species biofilms have become more and more in the focus of researchers. Wang et al. [51] investigated the adhesion of oral-relevant bacteria (*Streptococcus mutans*, *Streptococcus oralis*, *Actinomyces naeslundii*) in the absence and presence of a salivary-conditioning film. They found that *S. oralis* enzymatically degrades salivary conditioning film components to facilitate direct contact of other biofilm inhabitants with surfaces. Herewith, *S. mutans* can sense the underlying surface to define its appropriate adaptive response [51]. This fact can be seen as a new function of initial colonizers in multi-species oral biofilms.

**Figure 9:**
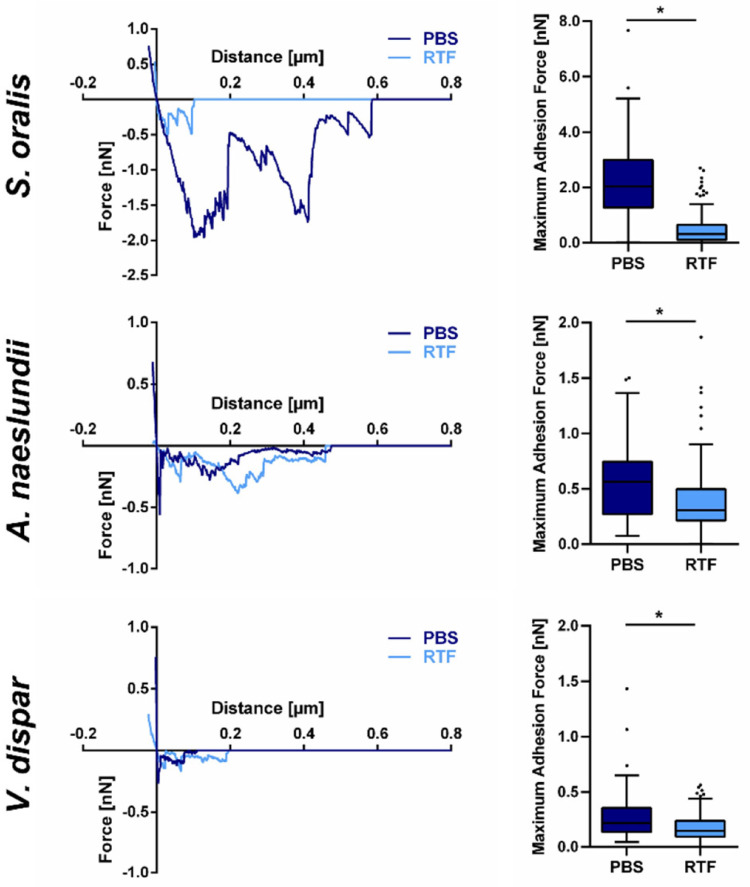
Exemplary force–distance curves (**left**) and single-cell adhesion forces (**right**) of oral bacteria (*Streptococcus oralis*, *Actinomyces naeslundii*, *Veillonella dispar*) on titanium in different buffers (PBS = phosphate-buffered saline; RTF = anaerobe-reduced transport fluid). * marks the significant difference in the adhesion forces as a function of the used buffer [52].

Doll-Nikutta et al. used the FluidFM^®^ technique to characterize the single-cell adhesion of oral bacteria to dental titanium. The authors identified distinct correlations between electrostatically driven adhesion forces, bacterial surface elasticity, surface charge, single-molecule attachment points, stretching capability, and metabolic activity. In addition, they evaluated the role of different oral bacteria in biofilm development. They found the strongest adhesion for pioneer commensals (*Streptococcus oralis* and *Actinomyces naeslundii*) and the lowest for secondary colonizers (*Veillonella dispar*) [52]. Exemplary results are shown in Figure 9.

Tu et al. provided a comprehensive review of the mechanism of bacterial adhesion to dental materials, exploring the relationship between surface properties and bacterial adhesion and the impact of bacterial adhesion on surface properties [74]. They summarized how surface properties influence oral biofilm formation and discussed possibilities for designing intelligent dental materials to reduce biological contamination. Tu et al. examined the impact of surface charge, hydrophobicity, hydrophilicity, and chemical properties of dental materials. They noted a need for more research on how surface stiffness and surface morphology affect bacterial adhesion and biofilm formation. Additionally, they concluded that dental materials and the acquired pellicle influence bacterial adhesion’s strength and mechanical stability [74]. Another crucial point highlighted by Tu et al. is the oral microbial composition, which significantly influences the development of oral diseases, on natural enamel and on dental restorative materials [74,75,76]. Regarding material properties, they asserted that negatively charged, superhydrophilic, superhydrophobic, and nano surfaces can all reduce bacterial adhesion. The scanning force microscope can investigate all these material parameters and their influence on bacterial adhesion.

Gunaratnam et al. delved deeper into the interaction between biological organisms and dental surfaces. They utilized single-cell force spectroscopy at the FluidFM^®^ to investigate the adhesion of yeast cells, specifically *Candida albicans*, to natural tooth enamel. *Candida albicans* are implicated in caries formation in children, initiating the colonization of teeth through the initial adhesion of individual yeast cells to the tooth enamel surface. Gunaratnam et al. focused on key adhesion parameters, including adhesion force, rupture length, and de-adhesion work of single saliva-treated and untreated yeast cells on tooth enamel, with and without a pellicle. Interestingly, they discovered increased adhesion parameters on the pellicle-covered enamel. This observation suggested that *C. albicans* likely utilize adsorbed ligands to bind to the tooth surface [77].

## 5. Lateral Force Microscopy

As previously described, SFM allows researchers to characterize dental materials at the nanometer scale and investigate the adhesion of various entities, including molecules, bacteria, and yeast cells, to dental surfaces. This fact offers valuable insights into the mechanisms underlying colonization and biofilm formation. Additionally, lateral force microscopy (LFM), also known as friction force microscopy, offers a complementary approach by enabling the measurement of friction coefficients, lubricating properties, and lateral detachment forces based on shear forces caused by flow.

**Figure 10:**
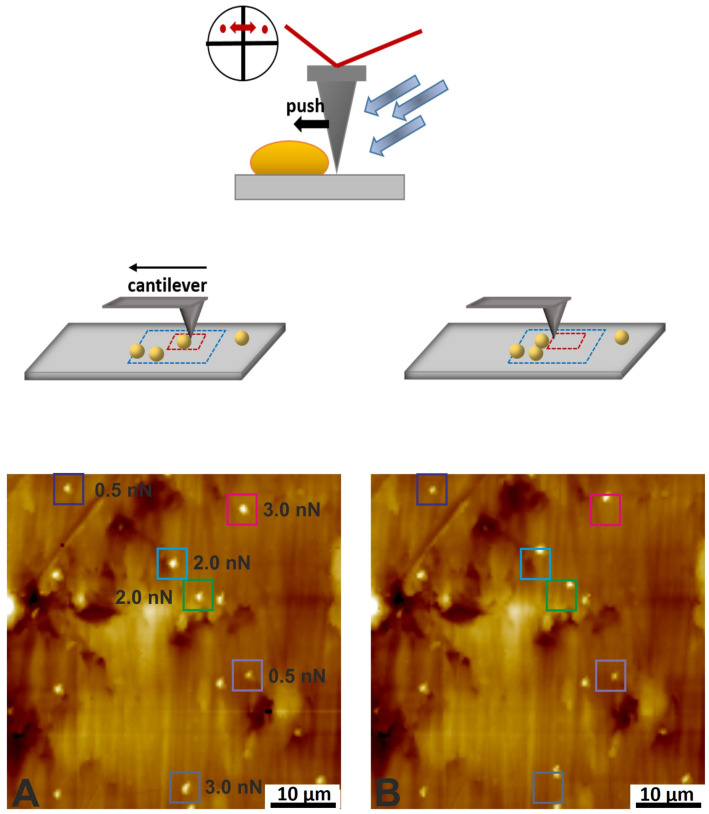
The cartoons show the single-cell lateral force microscopy measurement conduct (**top**). The SFM images before (**A**) and after the dislodgment test (**B**) to distinguish the bacterial displacement (**down**). Reproduced with permission from AVS [81].

Robinson et al. demonstrated the capability of LFM in revealing areas of differing friction force of maturation stage enamel crystals [19]. Jeng et al. explored the nanofriction properties of enamel rods using LFM, determining the coefficient of friction [40]. They observed that the friction coefficient parallel to the longitudinal axis of the rods is significantly lower than in the perpendicular direction. Sotres et al. utilized friction force spectroscopy to investigate the strength and structure of salivary films on model hydrophobic and hydrophilic substrates [78]. By varying the load and calculating the friction force, they differentiated two fractions of saliva-coated substrates with distinct abilities to diffuse along the substratum. Hahn-Berg et al. employed lateral force microscopy with colloidal probes to examine the lubricating properties between adsorbed salivary films [79]. They found that the presence of salivary pellicles between hard surfaces reduces the friction coefficient by a significant factor. To further understand the mechanism of salivary lubrication, Hahn-Berg et al. investigated the intraoral lubrication of three different key components of the pellicle using LFM [80]. In the context of bacterial cells, LFM provides access to the lateral detachment force. Huttenlochner et al. demonstrated using LFM to determine the lateral detachment force of single pre-adsorbed bacteria on titanium [81]. By applying lateral forces of varying magnitudes on single adherent cells and distinguishing between moved and unmoved bacteria, they established a relationship between surface parameters such as roughness, surface energy, zeta potential, and bacterial resistance to shear forces; see Figure 10.

## 6. Conclusions

This review provides an overview of SFM studies in dental research. Scanning force microscopy (SFM) emerges as a potent tool for imaging and characterizing dental materials at the nanoscale, offering unparalleled insights into their structure, properties, and interactions with biological systems. SFM boasts significant advantages, including generating high-resolution 3D images and operating in various environments such as air (including humidified conditions), liquid, or vacuum. Notably, SFM facilitates investigations in saliva or saliva-like environments, enhancing its applicability to dental research. The spectroscopic modes of SFM provide valuable access to interaction forces down to the pN regime, enriching our understanding of adhesive processes. Additionally, the development of FluidFM^®^ has expanded the capabilities of SFM, particularly in studying the interaction of biological cells (such as bacteria, spores, tissue cells, and yeast cells) with dental materials.

SFM-based measurements will continue to be pivotal in dental research, empowering researchers and practitioners to deepen their understanding of dental materials and their interactions with biopolymers and bacteria. This knowledge can drive the development of innovative solutions to enhance oral health and improve clinical outcomes in dentistry.
